# Association between 24-hour movement behaviors and depressive symptoms among urban older adults in china: a compositional isotemporal substitution analysis

**DOI:** 10.3389/fpsyt.2025.1706591

**Published:** 2025-12-12

**Authors:** Hu Ji, Glenn Roswal, Jing Min Liu, Yang Liu, Ya Qing Yuan

**Affiliations:** 1 Department of Physical Education, Shandong Jianzhu University, Shandong, Jinan, China; 2 School of Health Professions and Wellness, Jacksonville State University, Jacksonville, AL, United States; 3 Division of Sports Science and Physical Education, Tsinghua University, Beijing, China; 4 College of Sports and Health, Shandong Sport University, Shandong, Jinan, China

**Keywords:** 24-hour movement behaviors, time-use epidemiology, depression, compositional, isotemporal substitution, older adults, health promotion

## Abstract

**Objective:**

To examine the association between 24-hour movement behaviors and depressive symptoms in older adults using compositional data analysis, and to investigate the dose-response characteristics of time reallocations between movement behaviors in relation to depressive symptoms.

**Methods:**

A cross-sectional study was conducted among 1093 urban-dwelling older adults aged 60 years and above in selected communities of Jinan City, Shandong Province, China, between April 2024 and September 2024. The Chinese version of the International Physical Activity Questionnaire-Long Form (IPAQ-LF) was used to estimate time spent in moderate to vigorous-intensity physical activity (MVPA), light-intensity physical activity (LPA), sedentary behavior (SB), and sleep (SLP) across a typical 24-hour day. The Chinese version of the Patient Health Questionnaire Depression Scale-9 item (PHQ-9) was applied to assess depressive symptoms. Compositional isotemporal substitution models were employed to explore the associations between time reallocations among 24-hour movement behaviors and depressive symptoms, accounting for the co-dependent nature of time-use data.

**Results:**

(1) The geometric means of time spent in MVPA, LPA, SB, and SLP were 25.33 minutes, 141.26 minutes, 738.10 minutes, and 455.15 minutes, respectively. Variation matrix analysis revealed the highest log-ratio variance between MVPA and SB (0.168), and the lowest between SLP and SB (0.031). (2) The prevalence of screening-positive depressive symptoms was 16.29% among Chinese urban older adults. (3) Results from compositional linear regression models showed that time allocated to MVPA, LPA, and SLP (relative to the remaining movement behaviors) was negatively associated with depressive symptoms, while time spent in SB was positively associated. (4) Dose-response analysis further indicated that: (a) MVPA substitutions with other movement behaviors exhibited nonlinear and markedly asymmetric effects on depressive symptoms; (b) replacing MVPA with LPA, SB, or SLP resulted in increasingly larger changes in predicted scores as substitution duration increased, whereas the reverse substitution (MVPA for other movement behaviors) produced progressively smaller changes; and (c) substitutions between SB and LPA displayed linear and symmetrical effects.

**Conclusions:**

The findings provide evidence of an association between 24-hour movement behaviors and depressive symptoms in Chinese urban-dwelling older adults and reinforce the importance of achieving a balance between different types of movement behaviors over a 24-hour period for mental health.

## Introduction

In recent decades, the proportion of the older population has been increasing rapidly worldwide, primarily driven by longer life expectancies and declining fertility rates (1–3). According to the latest data from the World Health Organization, the global population aged over 60 has exceeded 1 billion, and this figure is projected to reach 1.4 billion by 2030 and 2.1 billion by 2050 (4). This growth is happening at an unprecedented pace and will further accelerate in the next decades, which presents considerable social, economic, and healthcare challenges, including increased demand for age-related medical services and long-term care facilities (5). Meanwhile, mental health disorders among older adults are becoming increasingly prevalent, creating significant challenges for individuals, their families, and healthcare systems (6–10). Among these mental health disorders in later life, depression stands out as one of the most prevalent and debilitating mental health issues facing older populations today. Depression not only substantially diminishes older adults’ quality of life but is also strongly associated with a range of adverse health outcomes. These include cognitive impairment, functional decline, and elevated suicide mortality rates (11–14). Addressing depression and its associated risk factors in older adults have therefore emerged as an urgent priority in public health.

A large number of studies have consistently confirmed that depression and its associated symptoms are independently linked to several unhealthy lifestyle movement behaviors, including physical inactivity (15, 16), extended periods of sedentary behavior (SB) (17–19), and inadequate or poor-quality sleep (SLP) patterns (20, 21). While these studies have yielded valuable insights, most of these studies have examined these movement behaviors in an isolation pattern. This approach overlooks the reality that physical activity (PA), SB, and SLP are co-dependent and mutually exclusive elements of the 24-hour day. Given the fixed nature of the 24-hour day, any increase in one movement behavior necessarily reduces time spent in others, and the resulting overall health impact often exceeds the sum of individual movement behavior effects when considered separately (22).

Failing to consider the compositional and interconnected nature of time-use movement behaviors can result in biased estimates and reduce the clarity of research findings (23). Given their combined influence and possible cumulative effects on mental health outcomes like depression, it is essential to investigate the integrated effects and interactions among these movement behaviors rather than focusing on any single movement behavior alone (24, 25). To appropriately address this co-dependence among time-use movement behaviors, a more suitable analytical approach is required. In recent years, compositional data analysis (CoDA) has emerged as a novel and effective statistical method designed specifically for multivariate data representing parts of a finite whole-such as time spent in SLP, SB, and PA, which together sum to the fixed 24-hour day (23, 26). Unlike conventional methods, CoDA explicitly respects the relative and co-dependent nature of time-use data, making it particularly well-suited for studies within the “24-hour movement behaviors” paradigm. Indeed, CoDA is increasingly recognized as the preferred analytical technique for such data (27), enabling a more comprehensive understanding of how daily movement behaviors collectively relate to health outcomes like depression.

Despite the acknowledged analytical advantages of CoDA, empirical evidence employing this innovative framework to investigate the association between movement behaviors and depression remains limited, particularly among older populations. Nonetheless, preliminary studies using CoDA have provided encouraging insights. For example, one cross-sectional European study involving Caucasian older adults demonstrated that reallocating 30 minutes from SLP to moderate to vigorous-intensity physical activity (MVPA) was significantly associated with a lower likelihood of depressive symptoms (28). Similarly, a recent study conducted among rural older adults in China also applied CoDA and identified significant associations between the reallocation of time among SLP, SB, and PA and depressive symptoms, further supporting the broad applicability and effectiveness of CoDA across diverse populations and settings (29). It is important to recognize that urban older adults differ significantly from their rural counterparts in various aspects, including lifestyle patterns, environmental factors (such as greater access to public parks, healthcare facilities, transportation options), socioeconomic status, as well as the availability and utilization of healthcare services. These contextual differences are likely to shape daily movement behaviors and influence related mental health outcomes (30–32). Consequently, investigating whether similar or distinct behavioral associations exist within urban populations is critically important.

Considering this research gap, the primary objective of the present study was to investigate the association between integrated 24-hour movement behaviors and depressive symptoms using compositional data analysis among a relatively large and representative sample of urban-dwelling older adults in China. Specifically, the study sought to generate urban-specific evidence and to examine whether associations observed in other settings also hold within urban contexts. Furthermore, this study aimed to identify optimal reallocations among SLP, SB, and PA that could effectively contribute to the reduction of depressive symptoms. Through such evidence-based insights, this research seeks to provide practical guidance for mental health promotion and targeted lifestyle interventions tailored specifically for aging urban populations, supporting healthier and more fulfilling lives among older adults.

## Methods

### Participants and procedure

A multistage cluster random sampling method was used to select study participants. First, one administrative district was randomly selected from the 12 administrative districts of Jinan City, Shandong Province, China. Within the selected district, one subdistrict was randomly chosen from its 14 subdistricts. Subsequently, four communities were randomly sampled from the selected subdistrict. Eligible residents in these communities were invited to participate. Inclusion criteria: (1) community-dwelling adults aged ≥ 60 years; (2) usual residence in the selected urban communities of Jinan City for ≥ 6 months; (3) able to communicate in Chinese and to complete study questionnaires independently or with minimal assistance. Exclusion criteria: (1) individuals diagnosed with severe neurological or psychiatric disorders that could impede valid data collection or compliance with study procedures (e.g., dementia, schizophrenia, or bipolar disorder); (2) individuals with cognitive or communication impairment sufficient to preclude understanding or completion of questionnaires and instructions; (3) individuals with physical limitations that preclude participation in physical activity (e.g., inability to ambulate independently, severe motor dysfunction, or physician-advised movement behaviors restriction); (4) individuals with an unwillingness to provide informed consent.

Between April 2024 and September 2024, a total of 1,269 individuals were invited to participate in the study and complete the assessment questionnaires. After excluding 176 participants due to low-quality questionnaire responses, 1,093 participants (86.13%) were retained for the final data analysis. Prior to formal data collection, the research team carefully recruited investigators with extensive experience in both mental and physical health fieldwork. These investigators underwent comprehensive training sessions that covered the study’s objectives, strategies for effective community engagement, proper administration of questionnaires, and the critical importance of adhering to standardized assessment procedures and research protocols. To raise public awareness and promote participation among older adults, outreach campaigns were conducted within the sampled communities using multiple channels, including posters, radio announcements, and WeChat (a popular social media platform in China). Individual appointments were then arranged according to the sampling list, during which investigators provided detailed explanations of the study’s goals, methods, procedures, and potential benefits. Subsequently, specific dates and times were scheduled for face-to-face, home-based interviews. Recognizing the sensitive nature of questions related to depressive symptoms, all assessments were conducted in private, one-on-one settings to ensure participant confidentiality and comfort.

### Ethical considerations

This study was reviewed and approved by the university’s ethics committee (Approval No. H20051S-2401). Written informed consent was obtained from all participants. All procedures conformed to the latest revision of the Declaration of Helsinki. (33).

### Measures

#### 24-hour movement behaviors

The 24-hour movement behaviors assessed in this study included PA, SB, and SLP time. These behaviors were measured using the Chinese version of the International Physical Activity Questionnaire long form (IPAQ-LC), which demonstrates acceptable reliability, with internal consistency coefficients (ICC) ranging from 0.79 to 0.87 (34, 35). Participants were asked to recall the number of days and the duration (in minutes per day) they engaged in three intensity levels of PA: light-intensity physical activity (LPA), moderate-intensity physical activity (MPA), and vigorous-intensity physical activity (VPA) over the previous 7 days. MVPA was defined as the sum of MPA and VPA. Weekly minutes for each PA intensity (LPA, MPA, VPA) were calculated as “reported days × minutes per day” and then converted to minutes/day by dividing by 7 days. For SB and SLP time, participants reported the average time (in hours and minutes per day) spent on these movement behaviors separately for weekdays and weekends during the past week. The daily time of SB and SLP was calculated by dividing the weekly time of SB and SLP by 7 days.

#### Depressive symptoms

The occurrence of depressive symptoms among older adults was evaluated using the Patient Health Questionnaire Depression Scale-9 item (PHQ-9) over the past two weeks (36, 37), which is a well-validated screening test for depression in this population (38–41). The PHQ-9 consists of nine items that measure the depressive state and severity of older adults, with each item rated on a 4-point Likert scale ranging from 0 (“not at all”) to 3 (“nearly every day”). The total score ranges from 0 to 27. Higher scores indicate more severe depressive symptoms. A total PHQ-9 score of ≥ 5 defined the presence of any depressive symptoms, with severity categorized as 5-9 (mild), 10-14 (moderate), 15-19 (moderately severe), and 20-27 (severe) (42). In addition, consistent with systematic reviews and meta-analyses identifying PHQ-9 ≥ 10 as a validated threshold for probable depression, a cut-off point of ≥ 10 was applied for the screening-positive prevalence in the present study (43). Given the community-screening design, no clinical diagnostic verification was performed. For missing data, records with ≥ 3 missing items were excluded from PHQ-9 analyses. If 1 or 2 items were missing, their values were imputed as the participant’s mean of the completed items (44–46). In this study, the Cronbach’s alpha coefficient for the PHQ-9 was 0.784, indicating acceptable internal consistency.

### Statistical analysis

Statistical analysis was conducted using SPSS (version 26.0, IBM Corp., Armonk, NY, USA) and R software (version 4.2.0, R Foundation for Statistical Computing, Vienna, Austria), and significance was set at *P* < 0.05. (1) Descriptive statistical analysis: The differences in PHQ-9 screening-positive prevalence among older adults according to gender, educational attainment, living status, and marital status were tested using the Pearson chi-square test. The central tendency of the 24-hour movement behaviors was described using compositional geometric means. The variability of these movement behaviors was assessed through pairwise log-ratio variance matrices. (2) Compositional multiple linear regression analysis: The “Clo” function from the “compositions” package was used to close the 24-hour movement behaviors data to a constant sum of 1440 minutes. Subsequently, the “ilr” function from the “robCompositions” package was employed to compute the isometric log-ratio (ilr) coordinates of the compositional data. The ilr-transformed movement behavior variables were treated as independent variables, while depressive symptoms were used as dependent variables. All models were adjusted for gender, educational attainment, living status, and marital status. (3) Compositional isotemporal substitution analysis: To more clearly illustrate the changes in predicted depressive symptom outcomes resulting from reallocations between movement behaviors, time reallocations were incrementally extended from −25 to +25 minutes in 5−minute intervals to explore the “dose-response” relationship between substitution duration and predicted depressive symptom outcomes.

## Results

Among the 1093 participants, the mean PHQ-9 score was 4.6 ± 5.4. As seen in **Table 1**, using the PHQ-9 ≥ 10, 16.29% (178/1093) were screening-positive for probable depression.

**Table 1:** Prevalence of PHQ-9 screening-positive cases (PHQ-9 ≥10) among older adults (N = 1093).

Item	Frequency and composition ratio	PHQ-9 screening-positive (≥10)		P
		Yes (n=178)	No (n=915)	
Gender				
Male	481 (44.01)	62 (34.83)	419 (45.79)	0.009
Female	612 (55.99)	116 (65.17)	496 (54.21)	
Age				
60-69	701 (64.14)	85 (47.57)	616 (67.32)	< 0.001
70-79	341 (31.20)	75 (42.13)	266 (29.07)	
80 or above	51 (4.66)	18 (10.12)	33 (3.61)	
Educational attainment				
Middle school or below	538 (49.12)	103 (57.86)	435 (47.54)	0.010
High school	472 (43.21)	69 (38.76)	403 (44.04)	
College or above	83 (7.67)	6 (3.38)	77 (8.42)	
Living status				
Living alone	208 (19.03)	72 (44.45)	136 (14.87)	< 0.001
Living together	885 (80.97)	106 (55.55)	779 (85.13)	
Marital status				
Married	871 (79.69)	113 (63.48)	758 (82.84)	< 0.001
Divorced/Widowed	222 (20.31)	65 (36.52)	157 (17.16)	

The results of the geometric means, as seen in **Table 2**, indicated that the time spent in MVPA, LPA, SB, and SLP was 25.33 minutes, 141.26 minutes, 738.10 minutes, and 455.15 minutes, respectively. In addition, variation matrix analysis revealed that the highest log-ratio variance was observed between MVPA and SB (0.168), indicating that these two movement behaviors were the least co-dependent. In contrast, the lowest log-ratio variance was found between SLP and SB (0.031), suggesting a strong co-dependence.

**Table 2:** Variation matrix and Geometric mean of data on SLP, SB, LPA and MVPA.

Movement behavior	SLP	SB	LPA	MVPA	Geometric mean (min/d)
SLP	0	0.031	0.054	0.087	455.15
SB	0.031	0	0.135	0.168	738.10
LPA	0.054	0.135	0	0.143	141.26
MVPA	0.087	0.168	0.143	0	25.33

SLP, sleep; SB, sedentary behavior; LPA, light-intensity physical activity; MVPA, moderate-to-vigorous-intensity physical activity. In a variation matrix, a value close to ‘0’ indicates a high proportionality between pairs of movement behaviors, whilst a value close to ‘1’ indicates the opposite.

Results from compositional linear regression models, as seen in **Table 3**, showed that time allocated to MVPA, LPA, and SLP (relative to the remaining movement behaviors) was negatively associated with depressive symptoms (*P* < 0.05), while time spent in SB was positively associated (*P* < 0.05).

**Table 3:** Multiple linear regression analysis with compositional between SLP, SB, LPA, MVPA, and depression.

Dependent variable	Independent variable	*β*	*P*	*Model P*	*R^2^*
Depression symptoms	SLP	-0.10	0.025	*P* < 0.001	0.415
	SB	0.78	*P* < 0.001		
	LPA	-0.21	0.034		
	MVPA	-0.63	*P* < 0.001		

SLP, sleep; SB, sedentary behavior; LPA, light-intensity physical activity; MVPA, moderate-to-vigorous-intensity physical activity.

**Figure 1:**
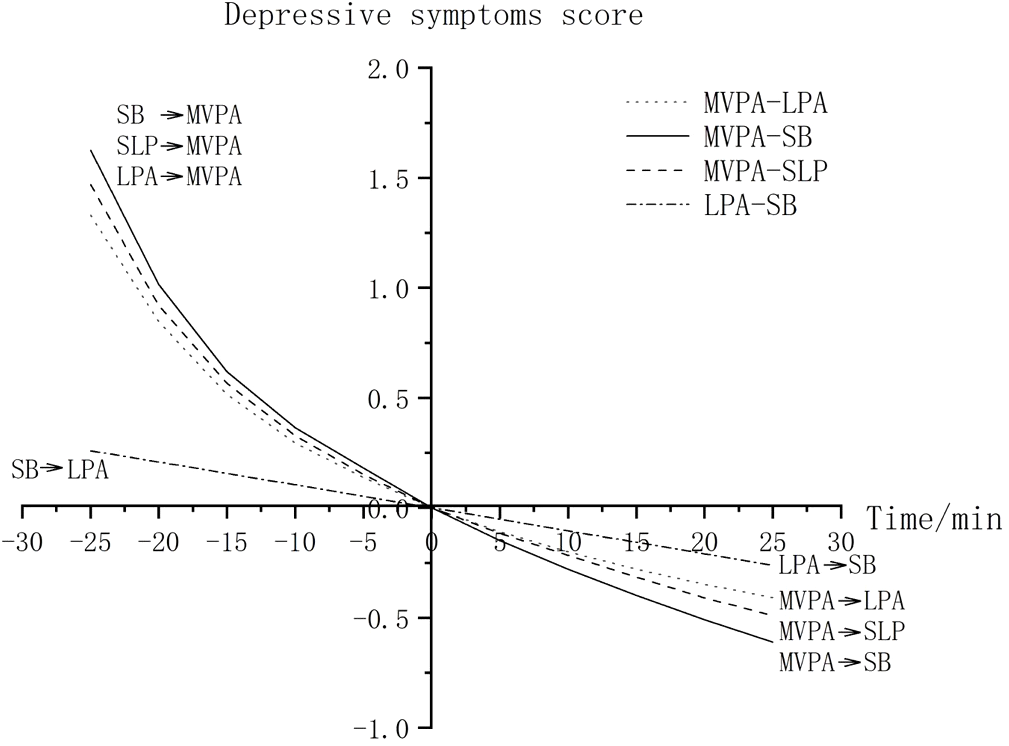
Impacts of mutual substitution between MVPA and LPA/SB/SLP, and between LPA and SB, on total depression score. SLP, sleep; SB, sedentary behavior; LPA, light-intensity physical activity; MVPA, moderate-to-vigorous-intensity physical activity.

Dose-response analysis further revealed that: (a) MVPA substitutions with other movement behaviors exhibited nonlinear and markedly asymmetric effects on depressive symptoms, (b) replacing MVPA with LPA, SB, or SLP resulted in increasingly larger changes in predicted scores as substitution duration increased, whereas the reverse substitution (MVPA for other movement behaviors) produced progressively smaller changes, and (c) substitutions between SB and LPA displayed linear and symmetrical effects (**Figure 1**).

## Discussions

The present study examined the cross-sectional relationships between 24-hour movement behaviors patterns and depressive symptoms among older adults residing in urban areas. The results indicated that 16.29% screened positive for probable depression of older adults in urban China experience depression, a rate that fell in the lower-middle range of previous studies reporting rates from 1.7% to 35.2% (47–49). This variability may arise from differences in diagnostic criteria, sample demographics (e.g., age, gender), assessment tools, or regional disparities. The urban focus of the current sample likely contributes to the relatively lower prevalence, as a meta-analysis by Zhang et al. (50) revealed that geriatric depression rates in urban China are generally lower than in rural areas. This discrepancy may be attributed to higher economic status, improved living conditions, greater access to healthcare resources, and stronger social networks in urban settings, which collectively act as protective factors against depression. For instance, urban environments often provide community centers that facilitate social engagement and mental health screenings, which may mitigate depressive symptoms. These findings underscore the importance of considering contextual and regional factors when interpreting depression prevalence in older populations. They also highlight the need for region-specific epidemiological studies and tailored mental health interventions, such as urban-specific mental health outreach programs, to address the unique challenges faced by older adults in different residential settings.

The results of this study demonstrated a strong association between 24-hour movement behaviors and depressive symptoms among older adults. Specifically, MVPA, LPA, and SLP were negatively correlated with depression levels, while SB was positively correlated. In other words, an increase in time allocated to MVPA, LPA, or SLP, accompanied by a corresponding reduction in time spent in other movement behaviors, was associated with lower levels of depressive symptoms. Conversely, increased SB time, at the expense of other movement behaviors, was linked to higher depression levels. Notably, the positive correlation between SB and depression underscores the detrimental effects of prolonged inactivity, which may exacerbate social isolation and reduce opportunities for mood-enhancing movement behaviors. These associations highlight the potential of optimizing the balance of movement behaviors to address depressive symptoms. The findings suggest that public health strategies aimed at reducing SB while promoting greater engagement in PA and ensuring adequate SLP could serve as effective, non-pharmacological approaches to reduce the risk of depression among older adults in urban settings.

The prior research by Kandola et al. and Xue et al. highlighted the powerful role of PA in mitigating depressive symptoms through diverse mechanisms (51, 52). Physiologically, PA regulates neurotransmitters, enhances neuroplasticity, balances hormone levels, and reduces inflammatory responses. Psychologically, it improves cognitive function, strengthens emotional regulation, fosters social interactions, and enhances self-efficacy. These mechanisms collectively underline PA’s critical role in protecting older adults from depression. Notably, Spirduso et al. emphasized that moderate PA positively influences mental well-being (53), while Gong et al. found that older adults with moderate PA levels have a lower risk of depressive symptoms compared to those with low PA (54). However, excessive PA may exacerbate physical strain in older adults with declining physical function, highlighting the need for moderation tailored to individual capacity. The present study’s finding that replacing SB with LPA reduces depression levels aligns with Kandola et al.’s observations (51). This suggests that the positive effects of LPA on depression should be emphasized while considering increasing PA time in older adults. Indeed, Füzéki et al. recommend that physically inactive individuals engage in activities of any intensity, given the proven health benefits of LPA (55). Thus, for older adults with insufficient MVPA and underlying chronic conditions or functional limitations that prevent them from participating in MVPA, increasing LPA offers a practical strategy to reduce depressive symptoms, particularly in urban settings where accessible activities like walking or tai chi are viable.

When SB was replaced by other movement behaviors, there was an overall trend of decreasing depression scores in older adults, with the strongest health effect when it was replaced by MVPA, suggesting that reducing the amount of time SB is used may be an important way to effectively intervene in older adults’ depression. This result is consistent with the study of Huang et al. and Kandola et al. (56, 57). Therefore, the present study suggests that increasing MVPA and decreasing SB is one of the most effective ways to reduce depression in older adults. However, the present study also revealed challenges in implementing MVPA-focused interventions, given the limited daily duration of MVPA among older adults and the high variance in the log-ratio of MVPA to other movement behaviors. This variability suggests that MVPA may not be easily scalable or uniformly achievable across all individuals. It may be particularly challenging for those with physical limitations or low baseline PA levels. Consequently, while increasing MVPA and decreasing SB remains one of the most effective strategies for improving depression outcomes, the practical constraints of MVPA adoption necessitate a broader approach. The positive effects of replacing SB with other movement behaviors, such as LPA or SLP, should not be overlooked, as these alternatives may be more feasible for certain subgroups of older adults. These findings underscore the need for flexible, individualized intervention designs that account for the heterogeneity in movement behavior preferences and capacities among urban older adults.

The current study found that SLP and SB were the most interchangeable movement behaviors among older adults, based on the log-ratio covariance matrix. Additionally, when SLP replaced SB, depression levels decreased in older adults. This result is consistent with findings from a previous study (57). It is important to note that both insufficient (< 7 hours) and excessive (> 8–9 hours) SLP duration represent risk factors for depression among older adults (19). Indeed, both SLP deprivation and prolonged SLP duration can lead to a cognitive system that is unable to better integrate emotional signals, which is prone to elicit negative emotions during emotion recognition, affective experience, and daily communication, and is more likely to increase the risk of developing depressive symptoms (58, 59). Our study found that the older adults spent about 7.5 hours on SLP, which lies within the commonly recommended 7–8 hours window. For those already within this range, the practical priority is to maintain adequate sleep while reducing SB and increasing MVPA/LPA. For individuals with short sleep, reallocating a portion of excess SB toward SLP to approach the 7–8 hours range may help improve emotional well-being. Practical measures include maintaining consistent sleep-wake schedules, improving SLP hygiene (e.g., reducing evening screen exposure and caffeine intake), creating a restful SLP environment despite urban noise, and incorporating relaxation techniques like mindfulness or light stretching. These strategies are particularly relevant for urban older adults, who may face unique challenges like technology overuse or environmental disturbances affecting SLP quality.

The dose-effect analysis revealed an asymmetry in depression score changes: reallocating time from MVPA to other movement behaviors gradually increased depression scores, while reallocating time to MVPA led to sharper decreases. This pattern, consistent with Olds et al. study (60), reflects the disproportionate time allocation across movement behaviors in the study sample, where SB averaged 10 hours daily and MVPA less than 0.5 hour. Reallocating 15 minutes from MVPA to other movement behaviors constitutes a relatively large proportion (approximately 50%) of its total duration, thereby producing a more substantial behavioral and psychological impact. In contrast, reallocating the same 15 minutes from SB represents only a small fraction (approximately 2.5%) of its total time, leading to a more limited effect on depression outcomes. These findings suggest that intervention strategies should not only aim to increase MVPA but also prioritize maintaining existing MVPA levels to prevent declines in mental health. Practical approaches to sustain MVPA could include structured community-based exercise programs, such as weekly brisk walking groups organized in urban parks. Additionally, integrating brief MVPA sessions (e.g., high keens running in place, stair climbing, square dancing) into daily routines at senior centers can help maintain PA levels. Collaborating with local healthcare providers to offer personalized PA plans, adjusted for individual mobility and health conditions, can also further ensure consistent MVPA engagement.

### Strengths

This study has a number of strengths. It is one of the first investigations with Chinese urban older adults to apply compositional data analysis (CoDA) together and compositional isotemporal substitution to examine 24-hour movement behaviors in relation to depressive symptoms. The sample size was relatively large, providing adequate power to detect associations and model substitution effects. We collected detailed self-reported time-use data encompassing PA across intensities, SB, and SLP, enabling a comprehensive 24-hour analysis. By using a CoDA approach, we properly accounted for the co-dependency of these movement behaviors and avoided the collinearity issues that arise from total-time constraints, which strengthens confidence in the findings. The isotemporal substitution modeling offers an intuitive, practical interpretation of the regression results by translating them into hypothetical movement behavior changes. Furthermore, we explored non-linear dose-response patterns, revealing insights about asymmetry that basic linear models would miss. These nuanced findings contribute to a deeper understanding of how movement behaviors collectively influence mental health in older adults.

### Limitations

Although this study has several strengths, there are several noteworthy limitations. First, the design was cross-sectional, meaning that all variables (movement behaviors and depressive symptoms) were measured at the same time. Therefore, we cannot establish causality or the direction of associations. It is equally plausible that having depressive symptoms lead to changes in movement behavior as those movement behavior patterns may influence depression. For example, an individual experiencing depressive symptoms might withdraw and become more sedentary and less active (reverse causation), rather than sedentariness causing the depression. Longitudinal studies are needed to untangle cause and effect and to see if changes in movement behaviors lead to subsequent changes in depressive symptoms. Second, the PHQ-9 captures depressive symptoms over only the past two weeks. As a screening tool, it does not establish clinical diagnoses and may under- or over-estimate long-term depression status. Accordingly, our results should be interpreted as associations with current symptom burden. Future work incorporating clinical interviews or longitudinal assessments is warranted. Third, reliance on self-reported data for SLP, SB, and PA may introduce measurement error and bias. Older adults might have difficulty accurately recalling or estimating their daily movement behaviors. Over or under-reporting is possible; for instance, social desirability could lead some to over-report PA or under-report SB time. SLP duration by self-report may not capture SLP quality or fragmentation. While the questionnaires were validated, they are inherently less precise than objective measures (e.g., accelerometers for PA or actigraphy for SLP). Misclassification of LPA versus SB (e.g., slow walking might be perceived as sitting by some) could attenuate true associations. Future research using wearable devices could provide more objective assessments of the composition of 24-hour movement behaviors. Fourth, unmeasured confounding is a concern. We adjusted for several covariates, but there may be other factors related to both movement behaviors and depressive symptoms that were not accounted for. For example, personality traits (e.g., low motivation or extraversion), social support and engagement, or diet and nutritional status might influence both an individual’s propensity to be active and their mental health. Chronic pain or subclinical illness could reduce movement behaviors and independently contribute to depression. We did not have detailed data on these variables, so residual confounding may partly explain the associations observed. Despite these limitations, the consistency of our results with theoretical expectations and prior studies increases confidence in the main conclusions.

## Conclusions

This study demonstrates an association between 24-hour movement behaviors and depressive symptoms in older adults living in urban China. The majority of older adults’ daily time is allocated to SB and SLP with relatively limited engagement in PA, reflecting a “more sitting, less moving” lifestyle pattern.

## Data Availability

The raw data supporting the conclusions of this article will be made available by the authors, without undue reservation.
